# Psychological and demographic characteristics of 368 patients with dissociative seizures: data from the CODES cohort

**DOI:** 10.1017/S0033291720001051

**Published:** 2021-10

**Authors:** Laura H. Goldstein, Emily J. Robinson, John D. C. Mellers, Jon Stone, Alan Carson, Trudie Chalder, Markus Reuber, Carole Eastwood, Sabine Landau, Paul McCrone, Michele Moore, Iris Mosweu, Joanna Murray, Iain Perdue, Izabela Pilecka, Mark P. Richardson, Nick Medford, on behalf of the CODES Study Group

**Affiliations:** 1 King's College London, Institute of Psychiatry, Psychology and Neuroscience, UK; 2 King's College London, School of Population Health and Environmental Sciences, UK; 3 South London and Maudsley NHS Foundation Trust, London, UK; 4 Centre for Clinical Brain Sciences, University of Edinburgh, UK; 5 Academic Neurology Unit, Royal Hallamshire Hospital, University of Sheffield, Sheffield, UK; 6 Centre for Social Justice and Global Responsibility, School of Law and Social Sciences, London South Bank University, London, UK

**Keywords:** Age at onset, dissociative seizures, functional impairment, psychopathology, quality of life

## Abstract

**Background:**

We examined demographic, clinical, and psychological characteristics of a large cohort (*n* = 368) of adults with dissociative seizures (DS) recruited to the CODES randomised controlled trial (RCT) and explored differences associated with age at onset of DS, gender, and DS semiology.

**Methods:**

Prior to randomisation within the CODES RCT, we collected demographic and clinical data on 368 participants. We assessed psychiatric comorbidity using the Mini-International Neuropsychiatric Interview (M.I.N.I.) and a screening measure of personality disorder and measured anxiety, depression, psychological distress, somatic symptom burden, emotional expression, functional impact of DS, avoidance behaviour, and quality of life. We undertook comparisons based on reported age at DS onset (<40 *v.* ⩾40), gender (male *v.* female), and DS semiology (predominantly hyperkinetic *v.* hypokinetic).

**Results:**

Our cohort was predominantly female (72%) and characterised by high levels of socio-economic deprivation. Two-thirds had predominantly hyperkinetic DS. Of the total, 69% had ⩾1 comorbid M.I.N.I. diagnosis (median number = 2), with agoraphobia being the most common concurrent diagnosis. Clinical levels of distress were reported by 86% and characteristics associated with maladaptive personality traits by 60%. Moderate-to-severe functional impairment, high levels of somatic symptoms, and impaired quality of life were also reported. Women had a younger age at DS onset than men.

**Conclusions:**

Our study highlights the burden of psychopathology and socio-economic deprivation in a large, heterogeneous cohort of patients with DS. The lack of clear differences based on gender, DS semiology and age at onset suggests these factors do not add substantially to the heterogeneity of the cohort.

## Introduction

Dissociative seizures (DS) are paroxysmal episodes of apparent altered responsiveness or transient loss of consciousness. They may be mistaken for epilepsy or syncope but are not accompanied by ictal encephalographic markers of epilepsy or physiological changes explaining unconsciousness or other associated paroxysmal behavioural and experiential alterations. The diagnostic gold standard is video-encephalographic (video-EEG) recording of typical events but it is widely recognised that proof of the diagnosis cannot always be obtained and that it may be entirely appropriate to commence treatment in the absence of diagnostic certainty (LaFrance, Baker, Duncan, Goldstein, & Reuber, 2013). Also referred to as ‘psychogenic non-epileptic seizures’, ‘non-epileptic seizures’, ‘Non-Epileptic Attack Disorder’ (NEAD), ‘functional seizures’ and the pejoratively-laden ‘pseudoseizures’, DS are classified as dissociative and functional neurological disorders within ICD-10 (World Health Organisation, 1992) and DSM-5 (American Psychiatric Association, 2013), respectively. The incidence of DS has recently been estimated as ~4.9/100 000/year (Duncan, Razvi, & Mulhern, 2011). Prevalence estimates vary from 2 to 50/100 000 (Benbadis & Hauser, 2000; Kanemoto et al., 2017).

Psychiatric comorbidities, such as depression, anxiety, personality disorder, and post-traumatic stress disorder are common in patients with DS (Bermeo-Ovalle & Kanner, 2018; Brown & Reuber, 2016). Reported rates vary across studies, possibly affected by inclusion criteria and assessment methods. Patients with DS often report comorbid somatic symptoms (e.g. pain) or additional functional neurological symptoms (McKenzie, Oto, Graham, & Duncan, 2011), and characteristically have an avoidant coping style and high levels of avoidance behaviour (Dimaro et al., 2014; Goldstein & Mellers, 2006; Goldstein, Drew, Mellers, Mitchell-O'Malley, & Oakley, 2000). Quality of life is lower in patients with DS than in those with epilepsy (e.g. Szaflarski et al., 2003). Long-term outcome, in terms of chronic disability and welfare dependency, is poor in about 70% of patients (Reuber, 2005) emphasising the need for evidence-based treatments (Martlew, Pulman, & Marson, 2014).

It is well accepted that most adults with DS are women (Gates, 2002). There are conflicting findings with respect to gender differences and psychiatric comorbidity of DS. Lower rates of psychiatric diagnosis and chronic pain (Thomas & Bujarski, 2013) and greater psychopathology (Holmes et al., 2001) have both been reported in men. Myers, Trobliger, Bortnik, & Lancman (2018) reported that men showed greater depression and a more avoidant coping style. In other research, no gender differences were found in current or previous use of mental health services and rates of other medically unexplained symptoms or panic attacks, although women were more likely to have a history of self-harm (Oto, Conway, McGonigal, Russell, & Duncan, 2005).

The onset of DS generally occurs before age 40 (Francis & Baker, 1999) but older people may develop the disorder nonetheless (Behrouz, Heriaud, & Benbadis, 2006; Duncan, Oto, Martin, & Pelosi, 2006). It has been suggested that there may be a different gender ratio and likely range of precipitant factors in older patients (Duncan et al., 2006). Patients developing DS over the age of 55 were more likely to be male, less likely to report previous sexual abuse, but more likely to report health-related traumatic experiences in one particular study (Duncan et al., 2006); in additon, there was a trend towards better mental health in the later onset subgroup. We have recently demonstrated that earlier age at DS onset is largely seen in women while men appear likely to develop DS at any age (Goldstein et al., 2019).

Approximately two-thirds of DS have a hyperkinetic semiology, and one-third hypokinetic (Meierkord, Will, Fish, & Shorvon, 1991; Reuber et al., 2003). Semiology is broadly comparable across cultures, genders, and age groups (Alessi & Valente, 2013; Asadi-Pooya & Emami, 2013; Korucuk, Gazioglu, Yildirim, Karaguzel, & Velioglu, 2018; Oto et al., 2005; Thomas & Bujarski, 2013; Wadwekar, Nair, Murgai, Thirunavukkarasu, & Thazhath, 2014). Convulsive DS may be more common in patients reporting antecedent sexual abuse (Selkirk, Duncan, Oto, & Pelosi, 2008).

Here we present the baseline characteristics of a large UK cohort of patients with DS recruited for a fully powered multi-centre, pragmatic, parallel arm, randomised controlled trial (RCT) to compare clinical and cost-effectiveness of Cognitive Behavioural Therapy (CBT) for patients with DS plus standardised medical care (SMC) *v.* SMC alone (Goldstein et al., 2015; Robinson et al., 2017). The large number of clinical centres involved in this prospective study avoids biases from smaller, single-centre studies. Some earlier measures on these patients have been reported elsewhere, along with all other patients who were initially consented to the screening phase of the RCT (Goldstein et al., 2019). We employed a wide range of measures in our RCT population, some of which had not previously been used in people with DS. Our current research questions were: (1) what are the demographic, clinical, and psychological characteristics of this large DS cohort?; (2) do their psychological characteristics differ according to: (i) whether age at onset of DS was before or after age 40; (ii) gender; or (iii) seizure semiology?

## Methods

### Recruitment into the study

Study recruitment was a two-stage process (Goldstein et al., 2015). Adult patients (⩾18 years old) with DS were initially recruited into a screening stage from neurology/specialist epilepsy clinics across 27 National Health Service (NHS) Trusts in England, Scotland and Wales. The main inclusion criteria were: the ability to give informed consent; DS occurring within the previous 8 weeks; DS confirmed by video-EEG telemetry or clinical consensus; no recorded history of intellectual disability; ability and willingness to complete seizure diaries and questionnaire measures; and willingness to be seen by a psychiatrist 3 months later. Main initial exclusion criteria were: concurrent active epilepsy; inability to complete seizure diaries or questionnaires; fulfilling DSM-IV (American Psychiatric Association, 1994) criteria for current drug/alcohol dependence; insufficiently fluent in English to complete questionnaires or attend CBT without an interpreter; or currently attending CBT for another condition that would not have finished prior to the CODES psychiatric assessment. We also excluded patients who had previously attended a CBT-based treatment for DS at a participating study centre.

During the screening stage, participants provided demographic information and then fortnightly data on DS frequency and severity, and were referred to the relevant study-affiliated psychiatry service (one of 17 NHS Neuropsychiatry or Liaison Psychiatry services) where, in addition to receiving a clinical psychiatric assessment (Goldstein et al., 2015), eligibility for the RCT was assessed. Criteria for trial eligibility (which, therefore, determined the cohort reported here) were as per the screening phase, plus these additional criteria: occurrence of DS in the 8 weeks preceding the psychiatric assessment; willingness to continue to complete seizure diaries and questionnaires, having provided regular data on DS frequency after receiving their diagnosis in the screening phase; and willingness to attend weekly/fortnightly CBT sessions. Randomisation had to be judged acceptable by the patient and clinician in each case. Active psychosis, imminent risk of self-harm, meeting DSM-IV criteria for current alcohol/drug dependence, current benzodiazepine use exceeding the equivalent of 10 mg diazepam/day, and existing diagnosis of factitious disorder were exclusion criteria at this stage. Eligible patients were consented; baseline measures, completed prior to randomisation, are reported in this paper.

**Fig. 1.**
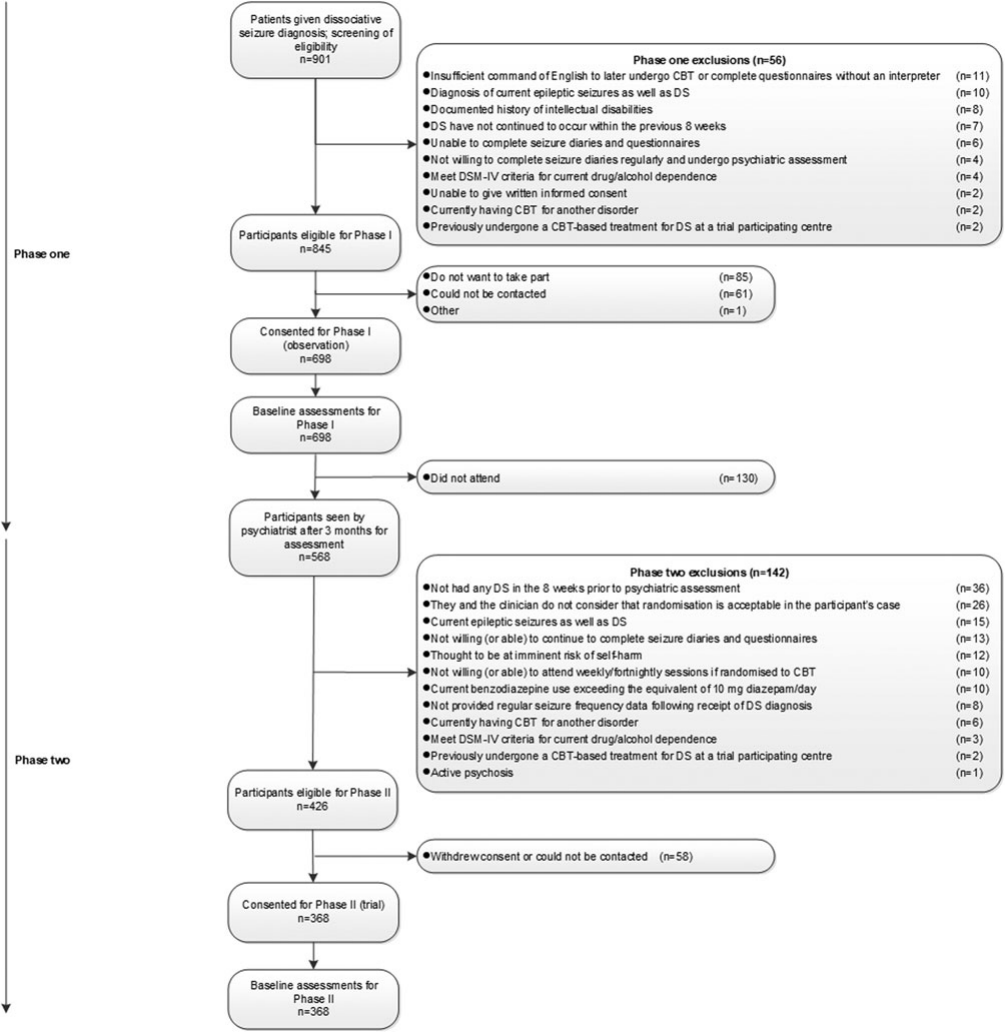
Study Flowchart up to randomisation. N.B. Characteristics of the 698 for whom baseline assessments were collected for Phase 1 have been reported by Goldstein et al. (2019). In addition, variables predicting which of the 698 participants did not attend psychiatry appointments have been reported by Stone et al. (2020).

Over the two stages of the study, participants were recruited between October 2014 and May 2017. Ethical approval for the study was granted by NRES Committee London-Camberwell St Giles (13/LO/1595). All participants gave written informed consent. Figure 1 shows the flow of participants through the two recruitment phases. Only the pre-randomisation data for the 368 who subsequently participated in the RCT are presented here.

### Measures

#### Demographics

We collected a wide range of clinical and demographic variables. Some of these measures were collected at consent to the screening phase of the overall study (Goldstein et al., 2019) and some were collected immediately pre-randomisation. We also recorded the Index of Multiple Deprivation (IMD) which gives a postcode-based measure of deprivation by locality, using the databases that were applicable when data collection commenced (Ministeries of Housing Communities and Local Government, 2010; Scottish Government, 2012; Welsh Government, 2011). These assess deprivation using weighted domains (seven for the English and Scottish IMDs, eight for the Welsh); here we report data separately for each region with IMD scores converted into quintiles.

Clinicians rated whether DS semiology was predominantly hyperkinetic or hypokinetic, and whether the person had a previous diagnosis of epilepsy, and if so: whether they still had epilepsy (but without having had a seizure in the past year); whether they now had DS alone; and whether the previous diagnosis of epilepsy might have been incorrect, or could not be verified from medical records.

Healthcare use and costs of this group will be considered elsewhere.

#### DS occurrence

The pre-randomisation measure of DS frequency was DS occurrence over the 4 weeks prior to psychiatric assessment (Robinson et al., 2017) with subjective severity of DS, and how bothersome they were, rated using the Seizure Severity Scale (Cramer, Baker, & Jacoby, 2002).

We administered measures to assess psychiatric comorbidities, psychological distress and symptoms, psychosocial functioning, heath-related quality of life, and beliefs about the DS diagnosis. These are described in detail in Table 1.

**Table 1.** Psychiatric and psychological assessment measures

Domain	Scale	Content
Psychiatric comorbidity	Mini-International Neuropsychiatric Interview (M.I.N.I. v6.0; (Sheehan et al., 1998)	Structured psychiatric diagnostic interview which requires mostly yes/no answers. Comprises modules corresponding to diagnostic categories standardised against DSM-IV; all but one (Antisocial Personality) are from Axis I.
	Standardised Assessment of Personality Abbreviated Scale, Self-Report (SAPAS-SR) (Germans et al., 2008).	8 questions ask about how the person sees themselves. Responses are yes/no; scores range from 0-8; scores of ⩾4 indicate the presence of personality disorder, which we interpret here as the presence of maladaptive personality traits. Fifty per cent of Germans et al.'s (2008) sample had received Axis II diagnoses on the SCID-II; the SAPAS-SR was found to correctly classify 81% of their sample. A three-factor structure was identified that broadly corresponded to Cluster A, B, and C personality disorders.
Psychological distress and somatic symptom burden	Generalised Anxiety Disorder Assessment (GAD-7) (Spitzer, Kroenke, Williams, and Lowe, 2006).	7- item scale used to measure anxiety. Good test-retest reliability and good criterion, construct, factorial and procedural validity. Higher scores indicate higher levels of anxiety. Cut-off of 10 identifies cases of GAD.
	Patient Health Questionnaire 9 (PHQ-9) (Kroenke, Spitzer, and Williams, 2001).	9-item scale used to measure depression. High internal consistency and high test-retest reliability. Scores range from 0 to 27. Higher scores indicate more severe depression. Scores of ⩾10 are classified as cases.
	Clinical Outcomes in Routine Evaluation (CORE-10) (Connell and Barkham, 2007).	10-item general measure of psychological distress (including risk). Good internal consistency; concurrent validity with other measures of depression, anxiety, and overall mental health. Higher scores reflect greater distress. Scores between 0 and 10 = non-clinical range; clinical range for distress is ⩾11.
	Modified PHQ-15 (Carson et al., 2015).	This asked participants whether they were “bothered a lot over the previous month” by a range of symptoms. The scale incorporated 15 common symptoms with which patients present in primary care, 10 ‘neurological’ symptoms and five psychological symptoms taken from the Prime MD Questionnaire. Higher total scores indicated greater numbers of comorbid symptoms; we also considered the frequency with which each symptom was reported.
	Beliefs About Emotions Scale (BES; Rimes and Chalder, 2010).	12-item scale measuring beliefs about the unacceptability of either expressing or experiencing negative emotions. The scale is reliable and valid. Each item is scored 0-6; total scores range from 0–72; higher scores indicate stronger beliefs about the unacceptability of negative emotions.
Psychosocial functioning and functional impact	Work and Social Adjustment Scale (WSAS) (Mundt, Marks, Shear, and Greist, 2002).	5-item scale measuring patients' self-reported perceptions of the functional impact DS had on their lives in the areas of work, home management, social leisure and private leisure activities, family and other relationships. Simple, valid measure of impaired functioning with high internal consistency and high test–retest reliability. Overall scores range from 0 to 40; higher scores indicate greater impact. Scores >20 reflect moderately severe impairment (or greater).
	Avoidance of People, Places, and Situations	Locally devised 3-item measure examining the avoidance of people, activities (e.g. physical exertion, bathing unsupervised) and situations (e.g. being out in public alone, social gatherings, using public transport) due to fear of having a seizure, each rated 0 (Never Avoid) to 10 (Always Avoid). Total possible score = 30. This scale has a Cronbach's alpha of 0.83, which shows good internal reliability between the three questions.
Health-related quality of life (HRQoL)	Short Form 12-item (version 2) Health Survey (SF-12v2) (Ware, Kosinski, Turner-Bowker, and Gandek, 2002).	12-item scale derived from the SF-36v2. Provides a measure of two overall physical and mental health concepts: the Physical Component Summary (PCS) and the Mental Component Summary (MCS). These have high internal consistency (Cheak-Zamora, Wyrwich, & McBride, 2009). A higher score on each measure indicates better HRQoL. Summary scores are norm-based T scores with an expected mean of 50 and standard deviation of 10.
	Visual analogue scale from EQ-5D-5L (EuroQol Research Foundation, 2009).	Person rates current health on a scale from 0 to 100; 0 = the worst health the person can imagine and 100 = the best health the person can imagine.
Beliefs about diagnosis	Beliefs about Diagnosis scale	Locally devised single item scale measuring participant's belief that they had been given the correct diagnosis of DS, on a single item scale from 0 (not at all) to 10 (extremely strongly).

### Statistical analysis

To describe our cohort of patients with DS, we summarised demographic, clinical, and psychological characteristics by using means (s.d.), medians (IQR) and frequencies (%), where appropriate. For clinical scales, established cut-offs and categories of severity were reported if they were available, as well as the overall scores. For the SAPAS-SR score, a bar graph was used to illustrate frequencies of responses according to Germans, Van Heck, Moran, and Hodiamont (2008)'s three personality trait clusters.

To address objective 2 we investigated univariate associations between age at onset of DS (<40 years *v.* ⩾40), gender (male *v.* female), and predominant seizure type (hypokinetic *v.* hyperkinetic), as well as associations between these binary variables separately and: current M.I.N.I. diagnoses (none *v.* any); previous M.I.N.I. diagnoses (none *v.* any); M.I.N.I. diagnosis of post-traumatic stress disorder (PTSD), a proxy measure for significant trauma history; scores on SAPAS-SR, CORE-10, GAD-7, PHQ-9, Modified PHQ-15, Beliefs About Emotions Scale, WSAS, and Avoidance of People, Places and Situations (total); and self-report of having previously sought psychiatric help (yes or no). In addition, for binary age at onset of DS (<40 *v.* ⩾40) only, we investigated differences in suicidality responses on the M.I.N.I. as a proxy measure of self-harm, and M.I.N.I. diagnosis of current panic disorder.

Formal group comparisons for continuous variables were carried out using *t* tests or Wilcoxon rank-sum tests, where appropriate. Namely, the WSAS and Avoidance scores violated the assumptions needed when running a *t* test, so the non-parametric rank-sum test was used instead. Fisher's exact test was used for all binary (categorical) variables. Each set of comparisons (i–iii) were adjusted for multiple testing by applying a conservative Bonferroni correction (significance level of 5%/number of tests performed to assess questions i–iii). For example, the 16 tests relating to 2(i) were compared to an adjusted *p* value of *p* < 0.003125 (0.05/16) to deem a significant result not a chance finding. Research question 2(ii) was addressed by 13 tests and 2(iii) consisted of 12 tests. *p* values have been reported prior to Bonferroni adjustment. All analyses were conducted in Stata version 15 (StataCorp, Texas).

## Results

### Demographic, clinical, and psychological characteristics of patients with DS

Table 2 presents descriptive summaries of demographic and clinical data for our cohort, with further variables summarised in Table 3. Of our cohort, 266 (72%) were women. Median age was 35 years, median age at DS onset was 29 years (IQR 19, 42), while modal age at onset was 19 years (three participants could not specify when their disorder had started). Median duration of DS disorder before diagnosis was 3 years (IQR 1, 8) although mean duration was 6.2 (s.d. 8.8) years.

**Table 2.** Demographic and clinical characteristics of the trial sample

	*n* (%)	*N* = 368
Age	Mean (s.d.)	37.5 (14.3)
	Median (IQR)	35 (25, 48)
	[range]	[18, 78]
Ethnicity	White	330 (89.7)
	Other (Asian, Black, Mixed or Other)	38 (10.3)
IMD score England (*n* *=* *284*)	1. Least deprived	31 (10.9)
	2	37 (13.0)
	3	54 (19.0)
	4	80 (28.2)
	5. Most deprived	82 (28.9)
IMD score Scotland (*n* *=* *77*)	1. Least deprived	10 (13.0)
	2	15 (19.5)
	3	13 (16.9)
	4	16 (20.8)
	5. Most deprived	23 (29.9)
IMD score Wales (*n* *=* *7*)	1. Least deprived	1 (14.3)
	2	0 (0.0)
	3	0 (0.0)
	4	1 (14.3)
	5. Most deprived	5 (71.4)
Qualifications (*n* *=* *367*)	None	43 (11.7)
	Secondary	89 (24.3)
	Vocational	120 (32.7)
	Further	56 (15.3)
	Higher	59 (16.1)
Current employment (*n* *=* *365*)	Employed or in education	123 (33.7)
	Not employed or in education	242 (66.3)
Receiving disability benefits if of working age (<65 years) and not working (*n* *=* *233*)	Yes	165 (70.8)
Receiving disability benefits if of working age (<65 years) and working (*n* *=* *110*)	Yes	18 (16.4)
Relationship status	Single	149 (40.5)
	Married/cohabiting	195 (53.0)
	Separated/divorced	19 (5.2)
	Widowed	5 (1.4)
Living arrangements	Alone	52 (14.1)
	With others	316 (85.9)
Has dependents	Yes	121 (32.9)
Relationship of dependent(s)^a^ (*n* *=* *121*)	Child	114 (94.2)
	Other (Partner, parent or other)	15 (12.5)
Has a carer	Yes	149 (40.5)
Relationship of carer(s)^a^ (*n* *=* *149*)	Partner	78 (52.3)
	Parent	35 (23.5)
	Child	17 (11.4)
	Other (Friend, paid or other)	52 (34.9)
Belief in diagnosis score (*n* *=* *366*)	Mean (s.d.)	8.0 (2.2)
	Median (IQR)	8 (7, 10)
	[range]	[0, 10]
Age at onset in years (*n* *=* *365*)	Mean (SD)	30.9 (14.1)
	Median (IQR)	29 (19, 42)
	[range]	[1, 76]
Years with DS (*n* *=* *365*)	Mean (SD)	6.2 (8.8)
	Median (IQR)	3 (1, 8)
	[range]	[0, 65]
Currently suffering from any other medical problem (*n* *=* *365*)	Yes	261 (71.5)
Previously diagnosed with epilepsy (self-report)	Yes	101 (27.4)
Currently prescribed anti-epileptic drugs	Yes	76 (20.7)
Monthly seizure frequency	Median (IQR)	15 (4, 47)
	[range]	[0, 649]
Longest period of time without DS in last 6 months (consecutive days) (*n* *=* *367*)	Median (IQR)	7 (2, 21)
	[range]	[0, 119]
Seizure severity	Mean (s.d.)	4.7 (1.6)
1 = not at all	Median (IQR)	5 (4, 6)
7 = very severe	[range]	[1, 7]
Seizure bothersomeness	Mean (s.d.)	5.3 (1.7)
1 = not at all	Median (IQR)	6 (4, 7)
7 = very bothersome		
	[range]	[1, 7]
SAPAS-SR *(n = 363)*	Maladaptive personality traits (⩾4)	211 (58.1)
CORE-10	Case (distress) (⩾11)	317 (86.1)
GAD-7	Case (anxiety) (⩾10)	191 (51.9)
PHQ-9 (*n* *=* *367*)	Case (depression) (⩾10)	236 (64.3)
SF-12v2 Physical Component Summary score (*n* *=* *366*)	Mean (s.d.)	39.7 (12.2)
	[range]	[13.4, 66.0]
SF-12v2 Mental Component Summary score (*n* *=* *366*)	Mean (s.d.)	37.8 (11.8)
	[range]	[13.4, 68.1]
EQ-5D-5L visual analogue scale (health today 1–100) (*n* *=* *363*)	Mean (s.d.)	55.5 (23.0)
	[range]	[1-100]

a Totals can add up to more than 100%.

**Table 3.** Descriptive summaries for age of onset, gender, seizure type, and psychological variables

	*n* (%)	*N* = 368
Age at onset of DS (*n* *=* *365*)	<40	264 (72.3)
	⩾40	101 (27.7)
Sex	Female	266 (72.3)
	Male	102 (27.7)
Predominant seizure type (*n* *=* *366*)	Hyperkinetic	236 (64.5)
	Hypokinetic	130 (35.5)
Current M.I.N.I. diagnosis	One or more	255 (69.3)
	None	113 (30.7)
Previous M.I.N.I. diagnosis	One or more	247 (67.1)
	None	121 (32.9)
SAPAS-SR score (maladaptive personality traits) (*n* *=* *363*)	Mean (s.d.)	3.9 (2.0)
	[range]	[0, 8]
CORE-10 score (distress)	Mean (s.d.)	18.2 (6.5)
	[range]	[4, 34]
GAD-7 score (anxiety)	Mean (s.d.)	9.8 (6.2)
	[range]	[0, 21]
PHQ-9 score (depression)	Mean (s.d.)	12.4 (6.6)
	[range]	[0, 27]
Modified PHQ-15 total (somatic symptoms)	Mean (s.d.)	16.7 (6.5)
	[range]	[2, 30]
Beliefs About Emotions score (*n* *=* *367*)	Mean (s.d.)	41.9 (16.9)
	[range]	[0, 72]
Work and Social Adjustment Scale (*n* *=* *366*)	Mean (s.d.)	22.7 (10.5)
	Median (IQR)	24 (15, 31)
	[range]	[0, 40]
Avoidance of People, Places, and Situations (total)	Mean (s.d.)	17.6 (9.0)
	Median (IQR)	20 (11, 25)
	[range]	[0, 30]
Ever sought medical help for a mental health problem	Yes	241 (65.5)
	No	127 (34.5)

In terms of IMD scores, over 50% of participants resided in areas falling in the two highest deprivation quintiles (57% of participants from England, 51% of those from Scotland, and 86% of those from Wales) as measured by an index of the general population. The majority (56%) had completed secondary or vocational education, two-thirds were not currently in employment or education and there were high rates of receipt of disability benefits. Of the total, 40% reported having a carer who, in just over half the cases, was their partner.

Median belief in the diagnosis of DS (measured at initial recruitment) was 8 (IQR 7, 10), indicating strong belief in the diagnosis.

The majority of participants (*n* = 195; 53%) had received their diagnosis on the basis of video-EEG. For cases where video-EEG was not involved, or where a consensus diagnosis had not already been obtained, a consensus rating was sought from one of two neurologists within the project team. In total, 236 (65%) participants had predominantly hyperkinetic, and 130 (36%) predominantly hypokinetic, DS; this information was not available for two participants. A total of 101 participants (27%) reported they had received a diagnosis of epilepsy and 76 (21%) that they were currently being prescribed AEDs. At a level only slightly below patients' self-report, clinicians judged that 89 (24%) participants had received a previous diagnosis of epilepsy, but felt that around half of these had been misdiagnosed, while for around an additional third of these they considered it was impossible to determine the validity of this earlier diagnosis.

The majority (241; 65%) indicated they had previously sought help for a mental health problem and, of the 365 responding, 261 (72%) reported currently suffering from another medical problem.

#### Psychiatric comorbidities

Data on M.I.N.I. diagnoses is shown in Table 4. In total, 255 participants (69%) had at least one current M.I.N.I. diagnosis, and 247 (67%) at least one previous M.I.N.I. diagnosis. The median number of current M.I.N.I. diagnoses was 2 (range 0–8) (see Supplementary Table 1 for more detail). The five most common current diagnoses (present in >20% of the sample; Table 3) were Agoraphobia (44.8%), Major Depressive Disorder (31%), Generalised Anxiety Disorder (29.3%), Post-traumatic Stress Disorder (23.4%), and Social Phobia (Social Anxiety Disorder) (20.4%). The most common past diagnosis was Major Depressive Disorder (52.4%). Of the total, 63% met criteria for suicidality and of these, 22.4% were judged high risk, although none were felt to be at imminent risk of self-harm by their study psychiatrist. A very small number were found to show substance dependence, not detected during the earlier assessment, on the M.I.N.I.

**Table 4.** Numbers and percentages of sample attaining diagnoses on the Mini-International Neuropsychiatric Interview

	*n* (%)	*N* = 368		*n* (%)	*N* = 368
Major depressive disorder (current)	Yes	114 (31.0)	Post-traumatic stress disorder (current)^a^	Yes	86 (23.4)
Major depressive disorder (past)	Yes	193 (52.4)	Alcohol dependence (current)	Yes	4 (1.1)
Suicidality^a^	Yes	232 (63.0)	Alcohol abuse (current)	Yes	5 (1.4)
Suicidality risk level (*n* *=* *232*)	Low (1-8)	151 (65.1)	Substance dependence (current)^b^	Yes	2 (0.5)
	Mod (9-16)	29 (12.5)	Substance abuse (current)^c^	Yes	1 (0.3)
	High (≥17)	52 (22.4)	Psychotic disorder (current)	Yes	10 (2.7)
Bipolar I disorder (current)	Yes	4 (1.1)	Psychotic disorder (lifetime)	Yes	21 (5.7)
Bipolar I disorder (past)	Yes	22 (6.0)	Mood disorder with psychotic features (lifetime)	Yes	19 (5.2)
Bipolar II disorder (current)	Yes	3 (0.8)	Mood disorder with psychotic features (current)	Yes	11 (3.0)
Bipolar II disorder (past)	Yes	10 (2.7)	Anorexia Nervosa (current)	Yes	0 (0.0)
Bipolar disorder NOS (current) (*n* *=* *367*)	Yes	3 (0.8)	Bulimia Nervosa (current)	Yes	13 (3.5)
Bipolar disorder NOS (past) (*n* *=* *366*)	Yes	6 (1.6)	Anorexia Nervosa [Binge eating/purging] (current)	Yes	0 (0.0)
Panic disorder (lifetime)	Yes	106 (28.8)	Generalised anxiety disorder (current)	Yes	108 (29.3)
Panic disorder (current)^a^	Yes	57 (15.5)	Antisocial personality disorder (lifetime)	Yes	16 (4.3)
Social Phobia [social anxiety disorder] (current)	Yes	75 (20.4)	Agoraphobia (current)	Yes	165 (44.8)
Obsessive compulsive disorder (current) (*n* *=* *367*)	Yes	34 (9.3)			

NOS = not otherwise specified.

a Used in analyses to test for associations (objectives 2i-iii).

b Substance = cannabis (*n* = 2).

c Substance = cannabis (*n* = 1).

On the SAPAS-SR (completed by *n* = 363) the mean score was 3.9 (s.d. 2.0; range 0–8) and 211 (58%) of the cohort had scores suggesting maladaptive personality traits. Online Supplementary Figure 1 shows the frequency of responses arranged in the three clusters identified by Germans et al. (2008) as broadly corresponding to Personality Clusters A, B, and C. Items most frequently endorsed were: being a worrier, dependence on others, and being a perfectionist (‘Cluster C’ items), whilst the least frequently endorsed were ‘Cluster A’ items (not trusting others, difficulty making and keeping friends, and being a loner).

#### Measures of psychological distress and somatic symptom burden

Table 3 shows scores on the CORE-10, GAD-7, PHQ-9 (with further classifications of levels of severity in online Supplementary Table 2) and the Modified PHQ-15. On the CORE-10, mean total scores indicated moderate distress although most scores (over 86%) fell in the range likely to indicate clinically relevant levels of distress (as scores from mild to severe are considered clinically meaningful). Mean PHQ-9 scores suggested moderate depression, with the majority (64%) classified as meeting the ‘caseness’ criterion. Mean GAD-7 scores were suggestive of moderate levels of anxiety but over half the sample were classified as ‘cases’.

**Fig. 2.**
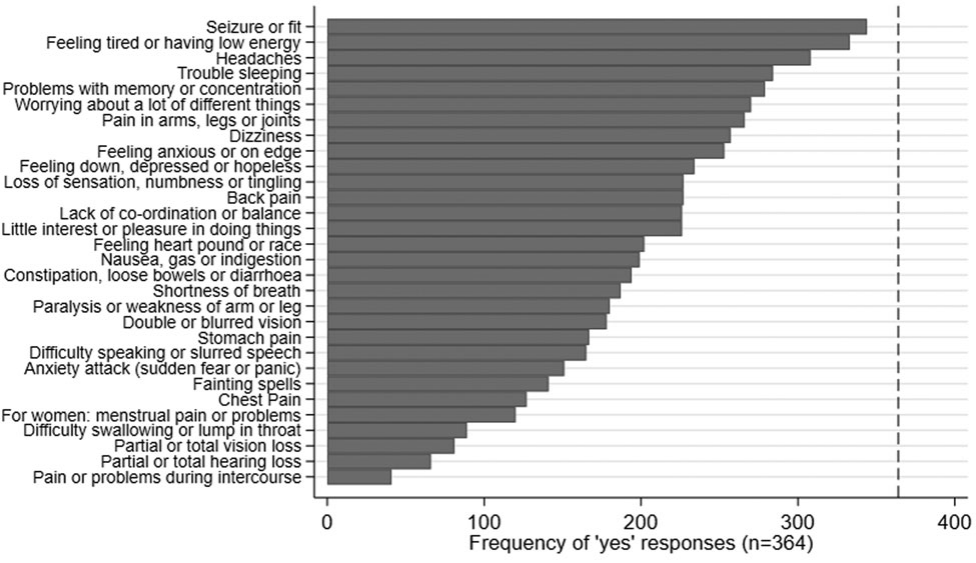
Occurrence of reported symptoms on the Modified PHQ-15. Occurrence of somatic symptoms reported on the Modified PHQ-15, illustrated in order of frequency of ‘yes’ responses: ‘During the past month have you been bothered a lot by [symptom]?’ The vertical dashed line indicates the number of participants who responded to the questionnaire overall (*n* = 364).

Figure 2 shows the reported frequency of Modified PHQ-15 symptoms. Unsurprisingly, DS occurrence was the most commonly reported symptom; the six next most commonly reported symptoms were tiredness/low energy, headaches, trouble sleeping, memory or concentration problems, generalised worrying and pain in limbs or joints.

The Beliefs About Emotions Scale data indicated that participants reported greater negative beliefs (mean 41.9; s.d. 16.9; *n* = 367) about the acceptability of experiencing and expressing negative thoughts and feelings than reported for a group of healthy controls comprising 67% females and mean age 37.6 years (mean score 27.9; s.d. 11.3; Rimes & Chalder, 2010).

#### Measures of psychosocial functioning and functional impact

The mean WSAS score was 22.7 (s.d. 10.5; *n* = 366), with a median of 24 (IQR 15–31). Scores on the Avoidance of People, Places, and Situations scale suggested slightly higher avoidance of situations and activities than of people, as a result of fearing seizure occurrence. The mean and median total scores for Avoidance were 17.6 (s.d. 9.0) and 20 (IQR 11–25), respectively, out of a possible 30.

#### Health-related quality of life

Summary scores on the SF-12v2 (*n* = 366) showed mean Physical Component Scores (PCS) of 39.7 (s.d. 12.2) and mean Mental Component Scores (MCS) of 37.8 (s.d. 11.8). The mean rating of current health on the EQ-5D-5L visual analogue scale (VAS) was 50.9 (s.d. 23.1).

### Differences in characteristics when age at onset <40 or ⩾40

Age at onset of DS was associated with gender: 77.7% (205/264) of women were <40 at the onset of DS compared to 58.4% (59/101) of men (*p* < 0.001). Older age at DS onset was associated with having no current M.I.N.I. diagnoses: 41.6% (42/101) of those ⩾40 at onset had none *v.* 26.5% (70/264) of those <40 (*p* = 0.007); and similarly for previous M.I.N.I. diagnoses: 40.6% (41/101) *v.* 29.5% (78/264). A further breakdown of number of M.I.N.I. diagnoses, split by age at onset, is given in online Supplementary Table 1. In addition, the median WSAS score for those aged <40 at onset of DS was 23 (IQR 14–30) compared to 27 (IQR 18–32) for those ⩾40; *p* = 0.0306. However, only the gender-specific differences remained significant after Bonferroni correction (*α* = 0.0031).

### Gender-specific differences in psychological characteristics

Men reported higher levels of avoidance behaviour and functional impairment compared to women. On the Avoidance of People, Places and Situations scale, the median total score for women was 18 (IQR 10–25; *n* = 266), and for men 22 (IQR 14–27; *n* = 102); *p* = 0.009 (Wilcoxon rank-sum). On the WSAS, the median score for women was 23 (IQR 14–30; *n* = 265) and for men 27 (IQR 17–33; *n* = 101); *p* = 0.042. Neither of these findings remained significant after adjusting for multiple testing (*α* = 0.0039).

### Differences in psychological characteristics relating to seizure semiology

There were no differences in psychological characteristics between patients with predominantly hyper- or hypokinetic DS.

## Discussion

We report data from 368 patients with DS recruited for a multi-centre RCT, allowing a detailed study of psychopathology and its relationship to demographic and clinical variables in a large, well-characterised cohort.

Although we did not collect detailed data about psychiatric comorbidity on the 698 people initially entering the study, basic demographics of the cohort (*N* = 368) reported here were similar to the larger group of 698 patients initially recruited into the CODES study (Goldstein et al., 2019). Furthermore, the generalisability of the sample reported here is supported by the observation that there were no significant differences in clinical and demographic variables between 568 of this initial group who attended a psychiatric assessment (and whose further eligibility was therefore considered for the RCT), compared to 130 who did not (Stone et al., 2020). In addition, we have recently shown (Goldstein et al., in press) that the 368 people reported here did not differ on key characteristics from the 58 people eligible for the RCT but who ultimately did not participate (see Fig. 1).

In the current cohort, median age at the onset was in adulthood although the distribution was skewed with modal onset at 19 years, with a duration of symptoms comparable to other studies (Asadi-Pooya & Sperling, 2015). Over half had received their diagnosis based on video-EEG recordings. Although around a quarter of the cohort had received a previous diagnosis of epilepsy, clinicians cast doubt on the accuracy of over 80% of these diagnoses, supporting previous accounts of frequent misdiagnosis in this patient group (Reuber, Fernandez, Bauer, Helmstaedter, & Elger, 2002).

Where diagnosis was not based on video-EEG but on clinical consensus, we acknowledge the possibility of misdiagnosis. In addition, we excluded participants with known epileptic seizures in the previous 12 months. Nevertheless, given the known limitations of diagnosis by seizure semiology (Syed et al., 2011) it is possible that some patients with active epilepsy were included despite our consensus review process, so the possibility of misdiagnoses is a limitation of the study.

Most of the patients lived in areas characterised by high levels of deprivation, extending previous findings from Scotland to the wider UK (Duncan, Oto, & Wainman-Lefley, 2012). Unemployment and economic inactivity were common. For those of working age but not working, almost three-quarters were in receipt of state financial disability benefits, a factor reported by others (McKenzie, Oto, Russell, Pelosi, & Duncan, 2010) to predict poor outcome. Interpretation of these findings is limited by the fact that our study did not allow us to compare our data with that from patients with other neurological or psychiatric conditions, and we cannot say whether other confounding factors might contribute to this pattern of socio-economic deprivation; nevertheless, at a population level, the findings demonstrate considerable socio-economic deprivation and benefit dependency in our sample, which may be relevant to access, uptake and response to interventions.

We acknowledge that the scales and questionnaires used in the study cannot be assumed to definitively diagnose psychiatric conditions, although they may strongly suggest them. Nevertheless, the consistently high scores seen across a range of measures suggest a high degree of psychiatric morbidity within the cohort, involving multiple different categories, primarily affective, anxiety, and personality disorders. Elsewhere, using an earlier version of the M.I.N.I. with DS patients, Mökleby et al. (2002) found a mean of 1.9 DSM-IV diagnoses with 91% of patients having at least two comorbid psychiatric diagnoses; our cohort had a similar mean of 1.9 (s.d. 1.9) and median of 2 (IQR 0–3) M.I.N.I. diagnoses. In our cohort 187/368 (51%) had two or more current M.I.N.I. diagnoses, most commonly anxiety disorders (i.e. agoraphobia, generalised anxiety disorder, social anxiety) as well as PTSD and depression, a profile similar to that reported by other studies (Bermeo-Ovalle & Kanner, 2018). However, the fact that some patients with more severe psychiatric comorbidities were excluded from the study on clinical grounds may mean that the rates of comorbidity in our sample are a little lower than in groups of DS patients seen in routine clinical practice.

Just under half the cohort reported symptoms suggestive of agoraphobia, which commonly develops in DS in relation to fear of having a seizure in public. Although standard psychiatric classification links agoraphobia to panic disorder, our data suggest that only 15.5% of the cohort would currently meet diagnostic criteria for the latter condition. This is about a third of the number with likely agoraphobia. This disparity is consistent with a model of dissociative seizures in which the seizures are viewed as a dissociative response to arousal in some ways analogous to panic attacks (Goldstein & Mellers, 2006). Such patients may have many features predisposing to panic disorder but since they do not experience panic *per se*, that diagnosis will not apply to them. Indeed, our previous work indicated that patients with DS experience autonomic symptoms of arousal in the absence of subjective awareness of feelings of panic (‘panic without panic’; Goldstein & Mellers, 2006).

Other symptom data suggest a prevalence of 31% for current major depressive disorder, and 23.4% for PTSD, within the range cited elsewhere (e.g. Bermeo-Ovalle & Kanner, 2018). In terms of questionnaire measures of psychological distress, between 52% and 86% of the sample were ‘cases’ (online Supplementary Table 1), depending on the specific measure, the highest value being for the most general measure, the CORE-10. Given the well-established association between DS and a history of trauma or abuse, a high rate of PTSD is perhaps unsurprising. However, in the experience of the authors, it is unusual for patients with DS to have been given a formal diagnosis of PTSD, even when they have had a specialist psychiatric assessment. This may be because the seizures themselves, and their psychosocial antecedents, tend to be the focus of assessment, such that other psychiatric symptoms may not be fully explored. The data also showed that 31% of patients had no concurrent psychiatric disorder detected on the M.I.N.I., emphasising the need to see other psychiatric comorbidities as common but not necessary for the development of DS.

On the SAPAS-SR, we found that 58% scored at or above the cutoff suggestive of maladaptive personality traits. We did not conduct clinical interviews to verify a diagnosis of personality disorder in this study, so cautious interpretation is needed here. Prior work suggests DS patients have higher rates of Cluster B personality disorders (antisocial, borderline, narcissistic, histrionic) than Cluster C (avoidant, dependent, obsessive-compulsive) disorders, with Cluster A disorders (schizoid, schizotypal, paranoid) least common (Bermeo-Ovalle & Kanner, 2018). Our sample most frequently endorsed items relating to worry and perfectionism, characteristic of Cluster C disorders, while least frequently endorsing items relating to difficulty making and keeping friends and being a loner. It may be that these results reflect the confounding effect of other psychiatric morbidity, in particular anxiety disorders which were common in our sample. Of note, the mean SAPAS-SR score in our study (3.9, s.d. 2.0) is comparable to that reported for a sample of patients with Generalised Anxiety Disorder (mean 3.8, s.d. 2.03; Mahoney, Hobbs, Newby, Williams, & Andrews, 2018). The Cluster C characteristics seen in our cohort are transdiagnostic processes which are targetable and could be included in future cognitive behavioural approaches.

Our cohort reported at least moderately severe functional impairment, similar to that reported elsewhere (Mayor et al., 2013), together with appreciable levels of avoidance of daily activities as a result of their DS, consistent with previous reports from our group and others (Goldstein & Mellers, 2006; Hixson, Balcer, Glosser, & French, 2006). Our participants also held particularly negative beliefs about experiencing and expressing emotions, with scores consistent with high levels of emotional avoidance. Taken together, these findings support our fear-avoidance therapeutic model for DS patients (Goldstein et al., 2015) and suggest that third wave psychotherapy could be an alternative to DS-specific CBT.

Health-related quality of life scores (SF-12v2) were low for both the physical and mental health composite scores, while a single rating of self-reported overall health from the EQ-5D-5L VAS suggested a particularly negative sense of general health, given that expected values in the UK are in the order of 80 (Janssen, Szende, Cabases, Vilagut, & König, 2019). Our participants reported a range of somatic symptoms on the Modified PHQ-15 which, with the exception of DS, were generally similar to those reported by a broader sample of neurology outpatients with symptoms unexplained by disease (Carson et al., 2015).

Our large sample allowed us to investigate the demographic and clinical correlates of psychiatric comorbidity. Overall, we identified a few gender- or age-related differences. Women were associated with an earlier age at onset of DS (<40) compared to men; this is the only finding that remains statistically significant after Bonferroni correction. Male participants demonstrated higher levels of avoidance behaviour and functional impairment due to their DS, while older age at onset patients had less severe psychopathology (fewer current M.I.N.I. diagnoses); however,these differences were no longer significant following correction for multiple testing. Thus, while there is a suggestion that older onset (and male) individuals with DS may have different psychological profiles, our findings fell short of statistical significance after using stricter adjustments than those used elsewhere (Duncan et al., 2006). We found no evidence for differences in psychological characteristics between patients with predominantly hyper- or hypokinetic DS.

In terms of limitations, we recognise that this study was conducted within the context of a large RCT and our inclusion and exclusion criteria may have selectively removed individuals with more severe comorbidity as well as those not wanting to be randomised. We excluded people with active epilepsy but some with inactive epilepsy did take part. Video-EEG was not uniformly used to diagnose patients, but the study was undertaken in the context of a pragmatic RCT and video-EEG is not uniformly/quickly available in all health service settings; if a consensus diagnosis had not already been obtained, a subsequent expert review of diagnosis was held when video-EEG had not been undertaken. Of note, the majority of patients held a strong belief in diagnosis, perhaps related to the verbal and written information relayed to patients in the study as part of the delivery of their diagnosis (Goldstein et al., 2015) which may not be typical of DS patients more widely.

The current study indicates the heterogeneity of psychopathology in a large UK sample of DS patients which may have implications for the outcome of a psychotherapeutic intervention, the findings of which will be reported elsewhere. Future consideration of the impact of such characteristics with respect to treatment will help to guide future treatment approaches and service provision in this area.

## Supplementary material

For supplementary material accompanying this paper visit https://doi.org/10.1017/S0033291720001051. click here to view supplementary material

## CODES study group

Anne-Mary Abe, Naghme Adab, Niruj Agrawal, Holger Allroggen, Debie Alvares, Thomasin Andrews, Heather Angus-Leppan, Julia Aram, Richard Armstrong, Antonio Atalaia, Manny Bagary, Maria Baldellou Lopez, Masha Bennett, Tamsin Black, Daniel Blackburn, Mayur Bodani, Mark Broadhurst, Alice Brockington, Elisa Bruno, Mary Buckley, Christine Burness, Hannah Callaghan, Richard Chalmers, Sam Chong, Muhammad Chowdhury, Fahmida Chowdury, Katia Cikurel, Giovanni Cocco, Hannah Cock, Sarah Cooper, Sarah Cope, Amy Copping, Elana Day, Robert Delamont, Gary Dennis, Christopher Derry, Rebecca Devlin, Jon M. Dickson, Beate Diehl, Clare Donnelly, Susan Duncan, Mark Edwards, Shan Ellawella, Cathy Ellis, Jennifer Elvish, Robert Elwes, Sandra Eriemo, Sofia Eriksson, Kerry Evans, Rafey Faruqui, Sarah Feehan, Gerald Finnerty, Lorena Flores, Nick Firth, Robert Fung, Paula Gardiner, Christopher Graham, Zayn Green-Thompson, Richard Grunewald, Robert Hadden, Khalid Hamandi, Rachel Harding, Sreedharan Harikrishnan, Simon Harrison, Helen Healy, Channa Hewamadduma, Stephen Higgins, Stephen Howell, Heloise Hunt, Abrar Hussain, Motchila Innocente, Graham Jensch, Michael Johnson, Harriet Jordan, Joanna Karlsson, Andrew Kelso, Steven Kemp, Jonathan Knibb, Norman Kock, Michael Koutroumanidis, Stjepana Kovac, Guru Kumar, Andy Laker, Guy Leschziner, Rebecca Liu, Dora Lozsadi, Lea Ludwig, Bridget MacDonald, Lindsey MacGregor, Melissa Maguire, Mark Manford, Davide Martino, Dougall McCorry, Annalisa McGorlick, Kenneth McKeown, Fiona McKevitt, Anna Meadow, Shafqat Memon, Ana Miorelli, Clinton Mitchell, Tejal N. Mitchell, Virginia Moffitt, Nicholas Moran, Aimee Morgan-Boon, John Moriarty, Marco Mula, Nandini Mullatti, Lina Nashef, Daniel O'Hara, Louise Oakley, Suzanne O'Sullivan, Lisa Page, Darshna Patel, Panayiota Petrochilos, Danielle Phoenix, William Pickerell, Thirza Pieters, Norman Poole, Gary Price, David Protheroe, Patrick Pullicino, James Purnell, Jennifer Quirk, Sanjeev Rajakulendran, Julie Read, Basil Ridha, Claire Rockliffe-Fidler, Carrie Rowbottom, Fergus Rugg-Gunn, Amrit Sachar, Romi Saha, Gerard Saldanha, Shanika Samarasekera, Violeta Sanchez Sanchez, Alastair Santhouse, Karen Scholes, Abhijeeth Shetty, Paul Shotbolt, Rachel Simkiss, Jasvinder Singh, Jananee Sivagnanasundaram, Sean Slaght, Philip Smith, Dilraj Sokhi, Biba Stanton, Liya Suvorova, Tayyeb Tahir, Ruth Taylor, Lara Teare, Lorenza Tedesco, James Teo, John Thorpe, Laura Toplis, Myrto Tsakopoulou, Ivona Tylova, Tracey Vick, Jane Vinnicombe, Matthew Walker, Cathy Walsh, Gillian Watson, Thomas Webb, Tim Wehner, Killian Welch, Kirstin Weyrich, Margaret Whittaker, Mirdhu Wickremaratchi, Louise Wicks, Mahinda Yogarajah.
