# Supplementary material for: Psychological and demographic characteristics of 368 patients with dissociative seizures: data from the CODES cohort
Source: Psychol Med. 2020 May 11;51(14):2433–45. doi: 10.1017/S0033291720001051 (PMC8506352; doi:10.1017/S0033291720001051)
Supplement: Supplementary file 1 [file S0033291720001051sup.zip › S0033291720001051sup001.docx]

Supplementary Table 1: Psychological and psychiatric measures by age at onset of DS (<40 vs ≥40)

| n (%) | <40 years at onset  (n=264) | ≥40 years at onset  (n=101) | Total  N=368 |
| --- | --- | --- | --- |
| Number of current MINI diagnoses  0  1  2  3  4  5  6  7  8 |  |  |  |
|  | 70 (26.5) | 42 (41.6) | 113 (30.7) |
|  | 54 (20.5) | 13 (12.9) | 68 (18.5) |
|  | 44 (16.7) | 13 (12.9) | 57 (15.5) |
|  | 40 (15.2) | 14 (13.9) | 54 (14.7) |
|  | 27 (10.2) | 8 (7.9) | 36 (9.8) |
|  | 18 (6.8) | 7 (6.9) | 25 (6.8) |
|  | 6 (2.3) | 4 (4.0) | 10 (2.7) |
|  | 2 (0.8) | 0 (0.0) | 2 (0.5) |
|  | 3 (1.1) | 0 (0.0) | 3 (0.8) |
| Number of previous MINI diagnoses  0  1  2  3  4 |  |  |  |
|  | 78 (29.5) | 41 (40.6) | 121 (32.9) |
|  | 110 (41.7) | 36 (35.6) | 146 (39.7) |
|  | 56 (21.2) | 21 (20.8) | 78 (21.2) |
|  | 15 (5.7) | 2 (2.0) | 17 (4.6) |
|  | 5 (1.9) | 1 (1.0) | 6 (1.6) |
| GAD-7  None (0-4)  Mild (5-9)  Moderate (10-14)  Severe (15-21) |  |  |  |
|  | 63 (23.9) | 32 (31.7) | 97 (26.4) |
|  | 58 (22.0) | 22 (21.8) | 80 (21.7) |
|  | 72 (27.3) | 20 (19.8) | 93 (25.3) |
|  | 71 (26.9) | 27 (26.7) | 98 (26.6) |
| PHQ-9  None (0-4)  Mild (5-9)  Moderate (10-14)  Mod-severe (15-19)  Severe (20-27) | *n=263* | *n=101* | *n=367* |
|  | 29 (11.0) | 17 (16.8) | 47 (12.8) |
|  | 61 (23.2) | 22 (21.8) | 84 (22.9) |
|  | 68 (25.9) | 20 (19.8) | 88 (24.0) |
|  | 59 (22.4) | 26 (25.7) | 85 (23.2) |
|  | 46 (17.5) | 16 (15.8) | 63 (17.2) |
| CORE-10  Healthy (0-4)  Low level (5-10)  Mild (11-14)  Moderate (15-19)  Mod-severe (20-24)  Severe (25+) |  |  |  |
|  | 2 (0.8) | 0 (0.0) | 2 (0.5) |
|  | 36 (13.6) | 13 (12.9) | 49 (13.3) |
|  | 39 (14.8) | 22 (21.8) | 63 (17.1) |
|  | 67 (25.4) | 30 (29.7) | 97 (26.4) |
|  | 64 (24.2) | 19 (18.8) | 84 (22.8) |
|  | 56 (21.2) | 17 (16.8) | 73 (19.8) |
