# Supplementary material for: Psychological and demographic characteristics of 368 patients with dissociative seizures: data from the CODES cohort
Source: Psychol Med. 2020 May 11;51(14):2433–45. doi: 10.1017/S0033291720001051 (PMC8506352; doi:10.1017/S0033291720001051)
Supplement: Supplementary file 1 [file S0033291720001051sup.zip › S0033291720001051sup002.docx]

**Supplementary Figure 1: Frequency of responses from the SAPAS-SR according to Germans et al’.s (2008) three factors**

*
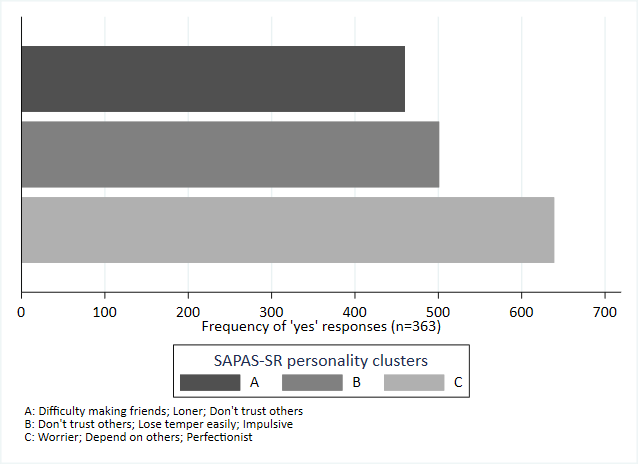
*
